# Evolutionary history of the C-repeat binding factor/dehydration-responsive element-binding 1 (CBF/DREB1) protein family in 43 plant species and characterization of CBF/DREB1 proteins in *Solanum tuberosum*

**DOI:** 10.1186/s12862-020-01710-8

**Published:** 2020-11-03

**Authors:** Wan Li, Yue Chen, Minghui Ye, Haibin Lu, Dongdong Wang, Qin Chen

**Affiliations:** 1 State Key Laboratory of Crop Stress Biology for Arid Areas, College of Agronomy, Northwest A&F University, Yangling, Shaanxi, 712100 China; 2 College of Food Science and Engineering, Northwest A&F University, Yangling, Shaanxi, 712100 China

**Keywords:** CBF/DREB1 family, Heat resistance, Cold resistance, Potato (*Solanum tuberosum*), Abiotic stress

## Abstract

**Background:**

Plants are easily affected by temperature variations, and high temperature (heat stress) and low temperature (cold stress) will lead to poor plant development and reduce crop yields. Therefore, it is very important to identify resistance genes for improving the ability of plants to resist heat stress or cold stress by using modern biotechnology. Members of the C-repeat binding factor/Dehydration responsive element-binding 1 (CBF/DREB1) protein family are related to the stress resistance of many plant species. These proteins affect the growth and development of plants and play vital roles during environmental stress (cold, heat, drought, salt, etc*.*). In this study, we identified CBF/DREB1 genes from 43 plant species (including algae, moss, ferns, gymnosperms, angiosperms) by using bioinformatic methods to clarify the characteristics of the CBF/DREB1 protein family members and their functions in potato under heat and cold stresses.

**Results:**

In this study, we identified 292 CBF/DREB1 proteins from 43 plant species. However, no CBF/DREB1 protein was found in algae, moss, ferns, or gymnosperms; members of this protein family exist only in angiosperms. Phylogenetic analysis of all the CBF/DREB1 proteins revealed five independent groups. Among them, the genes of group I do not exist in eudicots and are found only in monocots, indicating that these genes have a special effect on monocots. The analysis of motifs, gene duplication events, and the expression data from the PGSC website revealed the gene structures, evolutionary relationships, and expression patterns of the CBF/DREB1 proteins. In addition, analysis of the transcript levels of the 8 CBF/DREB1 genes in potato (*Solanum tuberosum*) under low-temperature and high-temperature stresses showed that these genes were related to temperature stresses. In particular, the expression levels of StCBF3 and StCBF4 in the leaves, stems, and roots significantly increased under high-temperature conditions, which suggested that StCBF3 and StCBF4 may be closely related to heat tolerance in potato.

**Conclusion:**

Overall, members of the CBF/DREB1 protein family exist only in angiosperms and plays an important role in the growth and development of plants. In addition, the CBF/DREB1 protein family is related to the heat and cold resistance of potato. Our research revealed the evolution of the CBF/DREB1 family, and is useful for studying the precise functions of the CBF/DREB1 proteins when the plants are developing and are under temperature stress.

## Background

Plants are continuously affected by various environmental factors such as temperature variation, high salinity, and drought during their growth process. These stresses can lead to many undesirable consequences, such as hindered plant growth and reduced crop yields. Plant responses to abiotic stress are complex and controlled by a variety of mechanisms, including physiological, biochemical, and molecular regulator mechanisms. Among them, molecular regulation may be related to stress perception, signal transduction, gene expression and final metabolic modification to prevent cell damage under abiotic stress [1].

Many genes related to plant stress resistance, such as those related to membrane stability [2], antifreeze protein genes [3], osmo-regulatory genes [4], and antioxidant enzyme activity genes [5], have been isolated, cloned and identified in plants. However, stress resistance is a quantitative trait controlled by multiple genes in plants, and it is difficult to cause significant effects by transferring only a single stress gene to plants [6]. Therefore, the study of transcription factors (TFs) that can improve the stress resistance of plants by regulating the expression of multiple stress-responsive genes has become a popular research.

The AP2/ERF superfamily is one of the largest TF families, and its members are involved in the regulation of growth and development and biotic and abiotic stress responses in plants [7, 8]. This family includes all the genes encoding at least one APETALA2 (AP2) domain and can be further divided into ethylene response factor (ERF), AP2, RAV (Related to ABI3/VP1), and soloists. Among these, the ERF family can be further divided into two subfamilies based on the amino acid sequence of their DNA binding domain: the CBF/DREB (C-repeat binding factor/Dehydration-responsive element-binding) subfamily (group A) and the ERF subfamily (group B) [9]. The DREB subfamily consists of six subgroups, of which the A1 subgroup includes CBF/DREB1 transcription factors [10]. The CBF/DREB1 gene is a type of plant-specific transcription factor that was first discovered in *Arabidopsis thaliana* (*Arabidopsis*) [11]. Members of the CBF/DREB1 family regulate the expression of most stress-inducible genes that are independent of the ABA pathway, and binds specifically to the CRT/DRE sequence (TACCGACAT) within the promoters of stress-inducible genes to activate the expression of the target genes, improving the resistance of plants to stress [12].

Members of the CBF/DREB1 subfamily exhibit different response patterns under various abiotic stresses, including low-temperature, drought, and high salinity [13–16], and transgenic methods using these genes have shown that these factors play important roles in abiotic stress signalling and tolerance [12]. In *Arabidopsis*, six CBF/DREB1 genes have been characterized [17]. Among them, AtCBF1, AtCBF2, and AtCBF3 are induced by cold stress, whereas AtCBF4, AtDREB1E, and AtDREB1F are induced by osmotic stresses such as drought and salt [10, 18–20]. The cold-resistance of tobacco (*Nicotiana tabacum*) and *Arabidopsis* can be enhanced because of the increased content of proline and soluble sugars by overexpressing the AtCBF3 gene [21, 22]. In rice (*Oryza sativa*), OsDREB1A is induced by salt and cold stresses [23, 24], whereas OsDREB1B and OsDREBL are induced by cold stress [25]. In *Arachis hypogaea*, PNDREB1 is strongly upregulated under low-temperature conditions [26]. The expression of PpCBF1 is also induced by NaCl, cold, drought, and ABA treatments in *Physcomitrella patens* [27]. Under cold stress, BjDREB1B and BoCBF/DREB1 are highly expressed in *Brassica juncea* and *Brassica oleracea* (kale), respectively [28, 29]. Overexpressing AtCBF3 in maize not only enhances cold tolerance, but also enhances drought and salt tolerance [30]. In transgenic *Arabidopsis*, salt tolerance and freezing tolerance are enhanced by overexpressing JcDREB from the woody biodiesel plant *Jatropha curcas* [31]. Overexpression of CkDREBa from *Caragana korshinski* confers drought and salinity tolerance to transgenic chrysanthemum [32]. MbDREB1 from dwarf apple (*Malus baccata*) increases plant tolerance to low temperature, drought, and salt stress via both ABA-dependent and ABA-independent pathways [33]. Transgenic *Arabidopsis* and rice plants overexpressing OsDREB1A are tolerant to low temperature, high salinity, and drought [13, 23]. Overexpression of CBF1/DREB1B in tomato (*Solanum lycopersicum*) enhanced the chilling and drought tolerance of transgenic tomato plants [34, 35].

Since release of the whole-genome sequences of increased numbers of plant species, many CBF/DREB1 transcription factors have been identified in species such as *Arabidopsis*, rice, wheat (*Triticum aestivum*), barley (*Hordeum vulgare*), and soybean (*Glycine max*) [10, 36–40]. However, no researchers have described a detailed and complete evolutionary history of these genes. In this study, we identified CBF/DREB1 genes from the complete genome sequences of 43 plant species (including lower plants and higher plants, such as algae, moss, ferns, gymnosperms, angiosperms) by using bioinformatic methods, such as analysis of phylogenetic relationships, motifs, gene duplications, gene characteristics and gene structure. In addition, potato is one of the most important crops species in the world, ranking fourth after rice, wheat and maize, and plays a significant economic role. As the main research object of our research team, we analysed Gene Ontology (GO) annotations and transcriptional profiles of the CBF/DREB1 proteins in potato. Based on the results, the evolution of the CBF/DREB1 genes can be revealed in high detail, and some useful information can be gained for further research on potato.

## Results

### Identification of CBF/DREB1 genes in 43 plant species

**Fig. 1:**
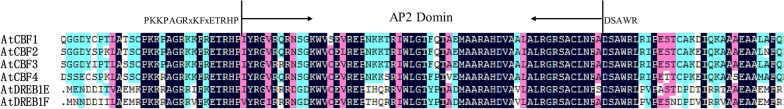
Amino acid sequence alignment of CBF/DREB1 proteins from *Arabidopsis thaliana*. The characteristic domains such as the PKK/RPAGRxKFxETRHP, DSAWR, and AP2 domains are indicated in the figure

To study the evolution of the CBF/DREB1 family, we used the CBF/DREB1 full-length protein sequence of *Arabidopsis* [17] as a query to search the available protein sequences from 42 other species, including algae, mosses, ferns, gymnosperms, and angiosperms. There are two conserved amino acid sequences, PKK/RPAGRxKFxETRHP and DSAWR upstream and downstream, respectively, of the AP2 domains of the CBF/DREB1 genes [41] (Fig. 1). Based on the sequence structural features and the domains of the CBF/DREB1 family, we removed incorrect and redundant sequences prior to further analysis of the remaining CBF/DREB1 sequences that could be considered putative genes. We ultimately identified a total of 292 proteins in 43 plant species and named them accordingly (Additional file 1a).

The number of CBF/DREB1 proteins ranged from 0 to 18 (Table 1). *Medicago truncatula* (Mt) and *Carica papaya* (Cp) contained 18 and 2 CBF/DREB1 proteins, respectively, which were greater and fewer than those in other plant species. Moreover, we obtained the genome length of 43 species from the NCBI database (Additional file 1a) and found that the number of CBF/DREB1 proteins was not related to the genome length.

**Table 1:** CBF/DREB1 proteins identified in 43 sequenced plant genomes

Lineage	Organism (Abbr.)	Numbers of CBF protein	Tandem duplication (pairs)	Segmental duplication (pairs)	Numbers of group I	Numbers of group II	Numbers of group III	Numbers of group IV	Numbers of group V
Algae									
	*Chlamydomonas reinhardtii* (Cr)	0	0	0	0	0	0	0	0
	*Volvox carteri* (Vc)	0	0	0	0	0	0	0	0
Mosses									
	*Marchantia polymorpha* (Mp)	0	0	0	0	0	0	0	0
	*Physcomitrella patens* (Ph)	0	0	0	0	0	0	0	0
Ferns									
	*Selaginella moellendorffii* (Sm)	0	0	0	0	0	0	0	0
Gymnosperms									
	*Picea abies* (Pa)	0	0	0	0	0	0	0	0
Angiosperms									
Amborellaceae	Amborella trichopoda* (Ar)	3	0	0	0	2	1	0	0
Eudicots									
Actinidiaceae	*Actinidia chinensis* (Ah)	9	0	2	0	2	0	1	6
Asteraceae	*Helianthus annuus* (Ha)	17	3	Not identified*	0	1	0	2	14
	*Lactuca sativa* (Ls)	13	2	Not identified	0	1	0	2	10
Brassicaceae	*Arabidopsis thaliana* (At)	6	1	2	0	0	0	4	2
	*Brassica oleracea* (Bo)	8	1	4	0	0	0	5	3
	*Brassica rapa* (Br)	9	0	8	0	0	0	5	4
	*Capsella rubella* (Cb)	6	2	2	0	0	0	4	2
Caricaceae	*Carica papaya* (Cp)	2	0	0	0	0	0	2	0
Chenopodiaceae	*Beta vulgaris* (Bv)	4	0	1	0	2	0	1	1
Cucurbitaceae	*Citrullus lanatus* (Cl)	3	0	1	0	0	0	3	0
	*Cucumis sativus* (Cs)	3	0	1	0	0	0	3	0
Euphorbiaceae	*Ricinus communis* (Rc)	4	0	1	0	1	0	3	0
Leguminosae	*Glycine max* (Gm)	11	0	20	0	4	4	0	3
	*Medicago truncatula* (Mt)	18	7	2	0	0	13	0	5
	*Phaseolus vulgaris* (Pv)	5	0	6	0	1	2	0	2
Malvaceae	*Eucalyptus grandis* (Eg)	15	9	3	0	0	13	0	2
	*Gossypium raimondii* (Gr)	8	0	7	0	3	0	5	0
	*Theobroma cacao* (Tc)	4	0	1	0	2	0	2	0
Nelumbonaceae	*Nelumbo nucifera* (Nn)	4	Not identified	0	0	2	0	2	0
Rosaceae	*Malus domestica* (Md)	4	0	Not identified	0	0	0	4	0
	*Prunus persica* (Pp)	6	2	2	0	0	0	6	0
	*Pyrus bretschneideri* (Pb)	11	Not identified	2	0	0	0	11	0
Rutaceae	*Citrus sinensis* (Ci)	3	0	0	0	0	0	3	0
Salicaceae	*Populus trichocarpa* (Pt)	5	0	0	0	1	0	4	0
Solanaceae	*Capsicum annuum* (Cu)	8	1	0	0	1	3	0	4
	*Solanum lycopersicum* (Sl)	7	1	1	0	1	3	0	3
	*Solanum tuberosum* (St)	8	1	0	0	0	4	0	4
Vitaceae	*Vitis vinifera* (Vv)	4	0	1	0	3	0	0	1
Monocots									
Arecaceae	*Elaeis guineensis* (El)	7	0	3	6	1	0	0	0
Musaceae	*Musa acuminate* (Ma)	8	0	8	4	4	0	0	0
Orchidaceae	*Phalaenopsis equestris* (Pe)	5	Not identified	0	4	1	0	0	0
Poaceae	*Brachypodium distachyon* (Bd)	16	3	0	16	0	0	0	0
	*Hordeum vulgare* (Hv)	15	0	Not identified	15	0	0	0	0
	*Oryza sativa* (Os)	10	0	3	9	1	0	0	0
	*Triticum aestivum* (Ta)	13	0	Not identified	13	0	0	0	0
	*Zea mays* (Zm)	10	0	3	9	1	0	0	0

^*^: The gene mapping information is unclear, it is impossible to determine the tandem duplication pairs; or segmental duplication information cannot obtained from the PGDD website

### Analysis of conserved motifs and gene duplication events

Prediction of protein motifs is an essential component of protein analysis. In this study, we used the MEME online tool to identify the motifs of 292 CBF/DREB1 proteins (the picture, which is not shown here, is too large to show clearly in the manuscript). (Additional files 2, 3). Twenty putative motifs were identified in these proteins. The motifs of the CBF/DREB1 family members were found to be conserved. The vast majority of proteins (more than 180 proteins) contained 8 or 9 motifs, and they were arranged in the same order. More than 80 proteins had 7 or 10 motifs, and a only few proteins had 5, 6 or 11 motifs. Additionally, OsCBF7 was the only protein with 4 motifs in rice (Additional file 1a).

To further understand the evolutionary relationship of CBF/DREB1 members, predicting of the molecular evolution rate can be used to clarify the gene evolution process [42]. In this study, we identified the tandem and segmental duplications in the 43 species (Table 1, Additional file 1 a, b), and the gene duplication events were identified in 28 species, based on the available information (in fact, due to incomplete information on gene location and on the PGDD website, we could not identify all the gene duplication events). In general, there were fewer tandem duplications than segmental duplication events. In addition, there were more CBF/DREB1 gene duplications in eudicots than in monocots. Most species only had tandem or segmental duplications, but some species had both. Tandem duplications did not occur in soybean, but we identified 20 segmental duplications, which was the highest number among all the proteins. *Eucalyptus grandis* contained the most tandem duplication events-9 pairs. We then calculated the Ka/Ks value for each duplication event (Additional file 1b), and the results showed that nearly every gene pair evolved with a Ka/Ks value less than one. However, in *Lactuca sativa*, the only gene pair had a Ka/Ks value of 1.1288.

### Analysis of the phylogenetic tree

**Fig. 2:**
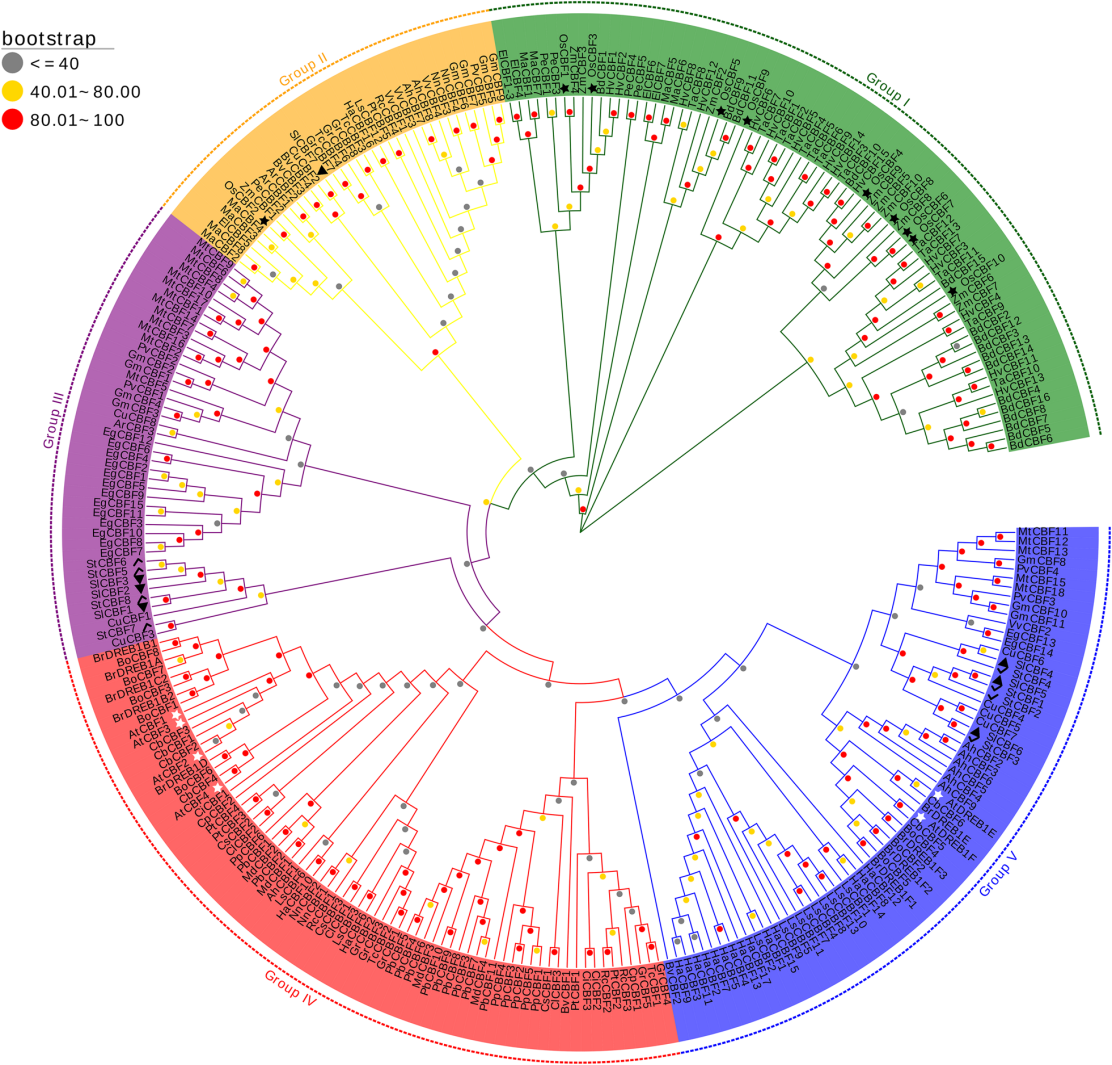
Neighbour-joining tree comprising CBF/DREB1 genes of 43 plant species. Bootstrap values were calculated in 1000 replications by using MEGA 7. The analysed CBF/DREB1 genes were distributed in five main groups (including Group I, Group II, Group III, Group IV and Group V), which were marked with different colours. Some CBF/DREB1 families are indicated with different marks. The black star indicates rice (*Oryza sativa*), the white stat indicates *Arabidopsis*, the black triangle indicates tomato (*Solanum lycopersicum*), and the black tick indicates potato (*Solanum tuberosum*). At the nodes, the gray dots indicate that the bootstrap values were less than or equal to 40; the yellow dots show that the bootstrap values were between 40 and 80, and the red dots represent bootstrap values that were greater than 80 but less than or equal to 100

A phylogenetic tree of the CBF/DREB1 members in the 43 plant species was constructed on the basis of the similarities of their protein sequences (Fig. 2). As shown in Fig. 2, the 292 identified CBF/DREB1 proteins from angiosperms could be roughly divided into five groups based on the phylogenetic tree. We analysed the distribution of the CBF/DREB1 proteins of the 43 species in different subgroups and found that the more closely related the species, the more similar the protein classification was (Table 1). Six CBF/DREB1 proteins in *Arabidopsis* were distributed in group IV and group V, which is consistent with the findings of previous studies [43, 44]. Group I and group IV had more members than did the other groups, each with more than 70 proteins. Group II contained the fewest CBF/DREB1 protein members. The CBF/DREB1 proteins in monocots are found only in groups I and II, and no group I CBF/DREB1 proteins were identified in eudicots. This suggested that the proteins of group I may have a unique role in monocots.

### Gene Ontology annotations and analysis of the RNA-sequencing data of StCBF proteins

**Fig. 3:**
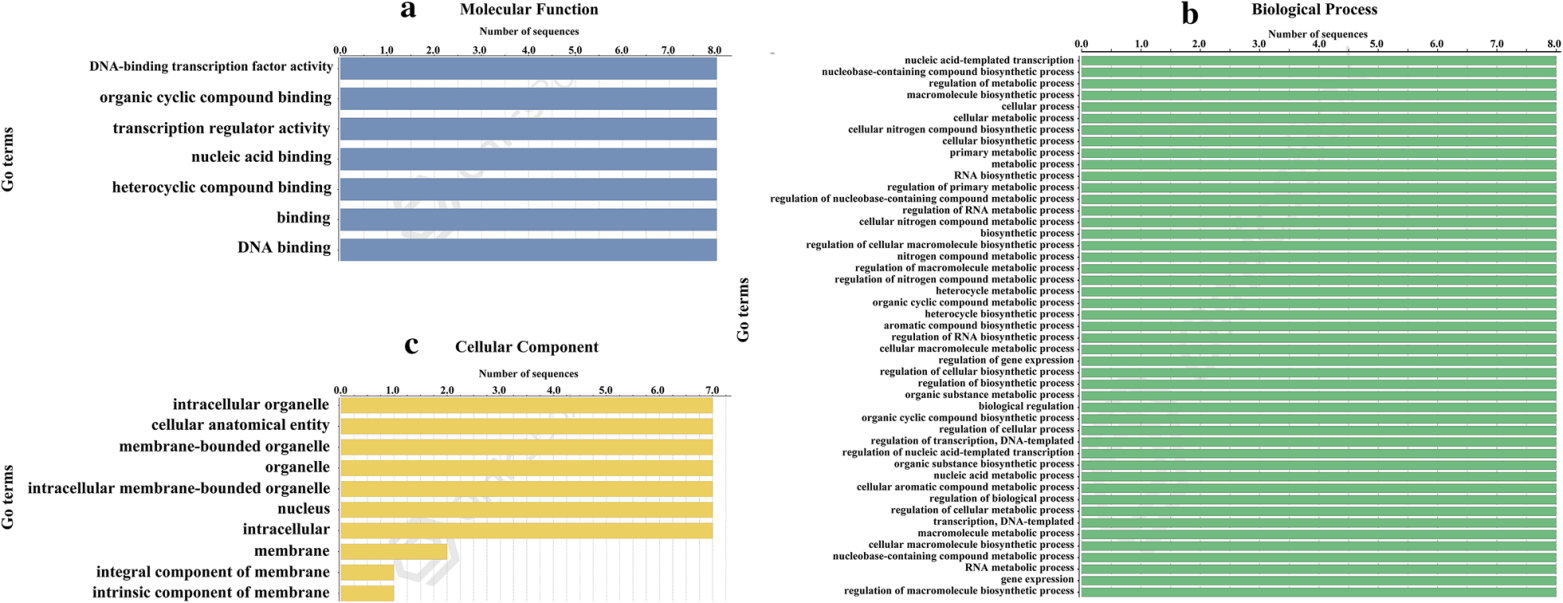
Information from Gene Ontology (GO) annotations. **a** Molecular function. **b** Biological process. **c** Cellular component. The numbers on the abscissa indicate the number of predicted proteins

To better understand the biological processes affected by CBF/DREB1 proteins, we selected the potato CBF/DREB1 proteins for GO analysis using the NCBI database [42] (Fig. 3, Additional file 4). In terms of molecular functions and biological processes, all eight genes had identical functions. These genes not only were involved in 7 molecular functions (such as DNA-binding transcription factor activity and transcription regulator activity), but also were involved in nearly 50 biological processes, including regulation of metabolic processes, RNA biosynthetic processes, regulation of cellular biosynthetic processes, etc. (Fig. 3a, b). This indicated that the StCBF genes play an important role in potato growth and development. In addition, predictions of the cellular components of the genes showed that, in addition to StCBF4, seven other genes were involved in component development of intracellular, organelle, membrane-bounded organelle, and so on. StCBF5 and StCBF6 appeared to be involved in the component development of the membrane, and StCBF5 was also involved in the integral and intrinsic components’ development of membrane (Fig. 3c, Additional file 4).

**Fig. 4:**
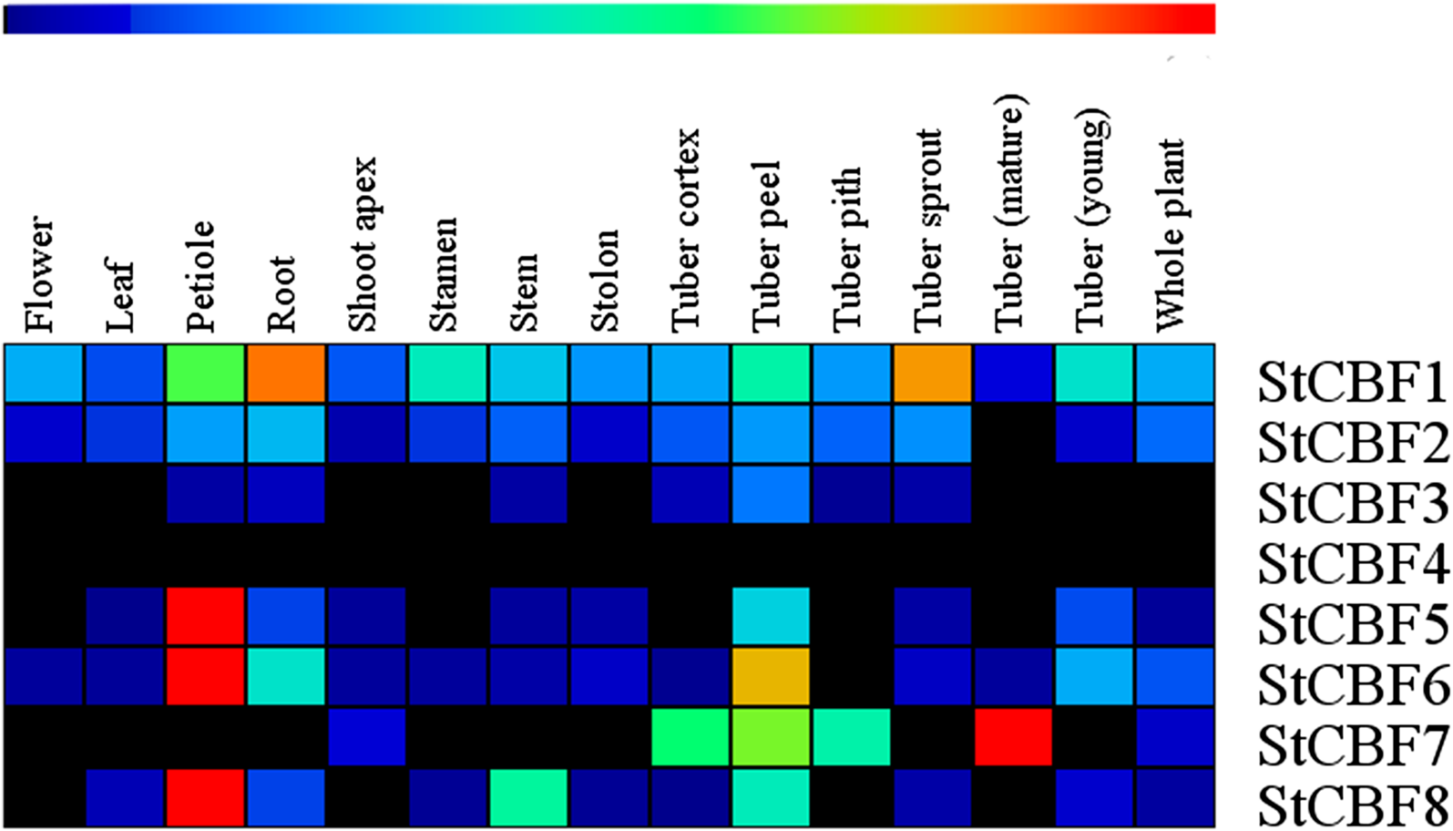
Heatmap based on the RNA-seq information of 14 different tissues of potato. The RNA-seq information was not processed. The heatmap shows CBF/DREB1 gene expression across 14 different tissues throughout the entire potato life cycle: including flower, leaf, stolon, petiole, root, young tuber, mature tuber, shoot apex, tuber peel, tuber cortex, tuber sprout, tuber pith, and stamen tissues. In the heat map, high expression is shown in red and low expression is shown in black

**Fig. 5:**
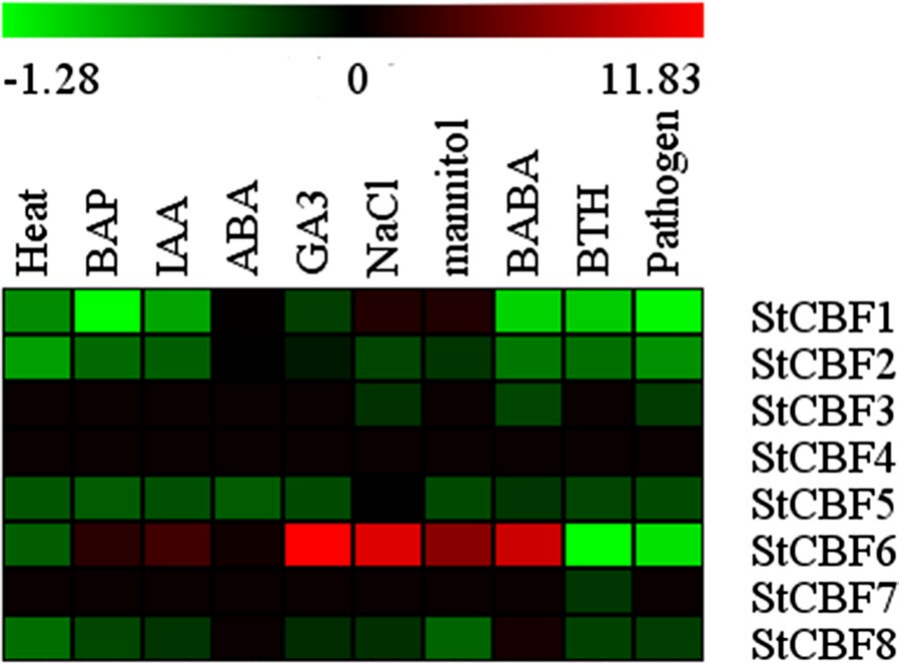
Expression profiles of 8 CBF/DREB1 genes under 10 different conditions in potato. Abiotic stress, biotic stress, and phytohormone treatment data and control data were obtained from the PGSC database. Transcripts were measured via RNA-Seq technology. The stress and hormone data were compared with the control data. In the heat map, upregulated expression is shown in red and downregulated expression is shown in green

We processed the RNA-seq database information and generated a heatmap (Figs. 4, 5). Figure 4 shows that the expression levels of the StCBF genes in all stages of potato development were not very high. Moreover, StCBF1, StCBF5, StCBF6 and StCBF8 were expressed in the petiole, and StCBF1 was clearly expressed in the root and tuber sprout. StCBF6 and StCBF7 were distributed in the tuber peel, and the expression of StCBF7 was detected in the mature tubers. The plants were treated with 10 different conditions, including phytohormone treatments (6-benzylaminopurine, BAP; indole-3-acetic acid, IAA; abscisic acid, ABA; gibberellic acid, GA3); abiotic stress (heat, drought, and salinity); and biotic stress (pathogen; DL-β-amino-n-butyric acid, BABA; acibenzolar-S-methyl, BTH) [45]. Under the 10 different treatments, most genes showed downregulated expression or little change in expression. However, in response to the BAP, IAA, GA3 NaCl (salinity), mannitol (drought) and BABA treatments, the expression of StCBF6 increased significantly. StCBF1 also increased slightly under drought, and salinity stresses. Under heat stress, no genes were upregulated, and few genes were upregulated in response to the other abiotic stress treatments (salt and drought).

### CBF/DREB1 gene expression profiles in response to temperature variations

**Fig. 6:**
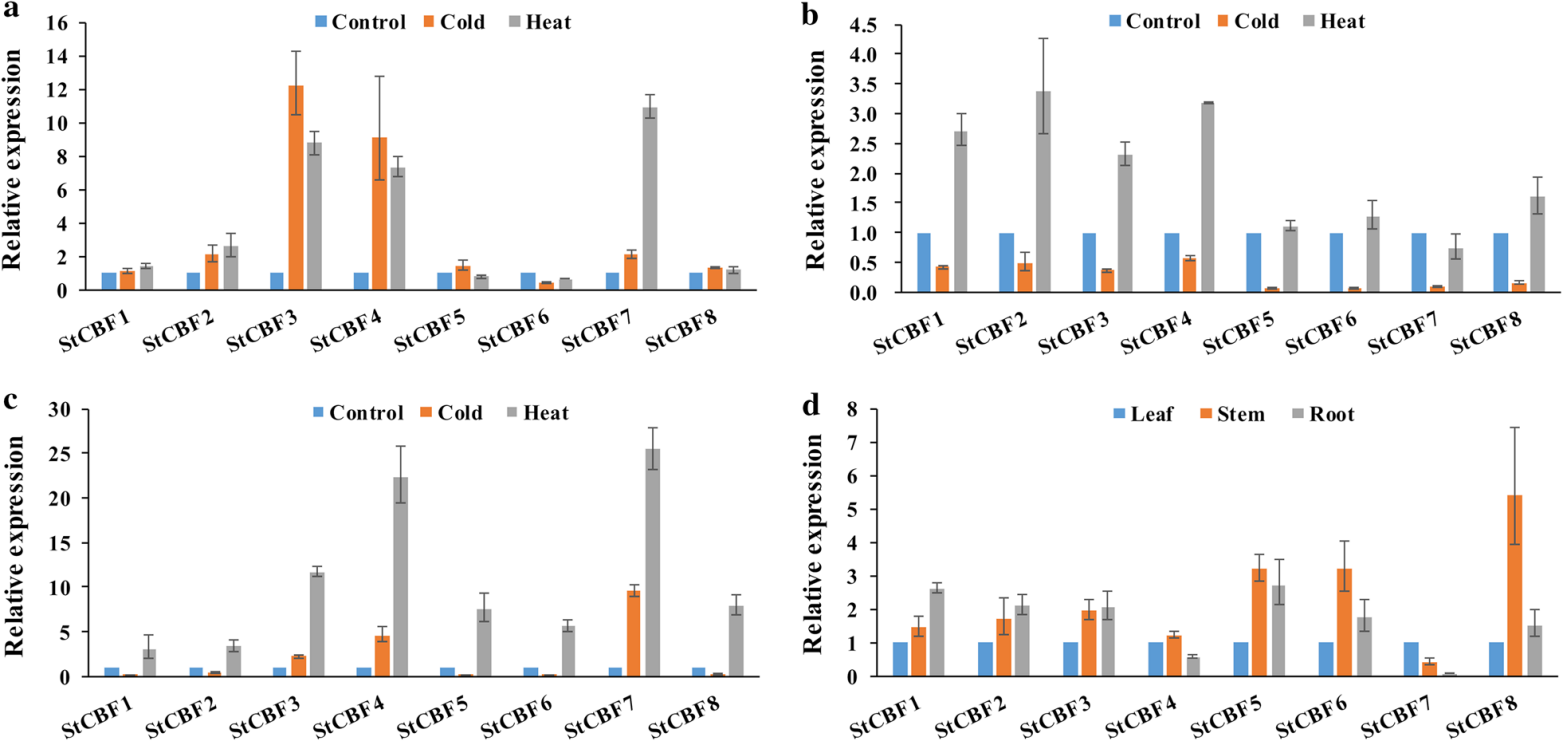
CBF/DREB1 gene expression in potato leaves, stems, and roots at 35 °C and 4 °C. **a** Leaf, **b** Stem, **c** Root, **d** Comparison of gene expression in the three tissues based on that in the leaves. The X-axes show 8 representative CBF/DREB1 genes, and the y-axes show scales of relative expression levels. The Ef1a gene was used as a reference transcript. Leaf, stem, and root tissues were sampled from the same parts of control and experimental plants. The quantitative data were measured by taking three biological replicates and two technical replicates, and the relative expression level of each gene was calculated using the 2^−ΔΔCt^ method

To demonstrate the change in CBF/DREB1 protein accumulation in different tissues (leaf, stem, and root tissues) in potato under low-temperature (4 °C) and high-temperature (35 °C) treatments, qRT-PCR analysis was used to conduct expression analysis (Fig. 6, Additional file 5). We mainly focused on the CBF/DREB1 genes of potato.

In the leaves (Fig. 6a), under low-temperature and high-temperature stresses, the expression levels of StCBF1, StCBF2, StCBF5, StCBF6, and StCBF8 did not change much, but the expression levels of StCBF3 and StCBF4 increased significantly. The expression of StCBF7 increased under high-temperature conditions. In the stems (Fig. 6b), the transcript levels of all genes decreased under low-temperature stress. However, notable upregulation of 4 genes (StCBF1-4) occurred under high temperature conditions, and the expression of StCBF7 decreased slightly, the expression of the other three genes did not significantly increase. In the roots (Fig. 6c), the expression levels of 8 genes increased significantly under high temperature. Under low-temperature conditions, the expression levels of StCBF4 and StCBF7 obviously increased, while the expression levels of StCBF3 barely increased, and the expression levels of other genes showed a decreased.

In addition, on the basis of the expression of genes in the leaves under normal conditions, we compared the expression levels of the 8 aforementioned genes in the 3 tissues (Fig. 6d). Except for StCBF7, the expression levels of 7 genes in the stems were higher than those in the leaves, and the transcript levels of StCBF4 and StCBF7 in the roots were lower than those in the leaves. Taken together, the results showed that StCBF genes in potato were expressed more in the roots and stems and less in the leaves, which is consistent with the data obtained from the PGSC website (Additional file 6).

## Discussion

CBF/DREB1 proteins have been identified in many species, such as wheat, rice, Chinese cabbage (*Brassica rapa*), and kale [43, 44, 46–50]. We combined these results, and we searched the CBF/DREB1 family again using the methods described in this article. For example, we found that Bra010463 (BrDREB1C1) [43] did not contain the PKK/RPAGRxKFxETRHP signature sequence, so we omitted it. In addition, we found that no CBF/DREB1 proteins were identified in algae, mosses, ferns, and the gymnosperms *Picea abies*. CBF/DREB1 proteins were identified only in angiosperms. According to the evolutionary relationships, the CBF/DREB1 protein did not evolve until the appearance of angiosperms.

The motifs of 292 identified proteins were analysed by the MEME website (Additional file 2). Previous studies have shown that certain conserved motifs to play functional and/or structural roles in active proteins [51]. Most proteins had 7–10 motifs, but OsCBF7 (LOC_Os06g06970.1, OsDREB1D) had only 4 motifs, which was significantly different from the numbers in the other proteins. Research has shown that OsCBF7 is a unique DREB homologous gene, and its expression cannot be detected with or without any stress in rice [23]. Yeast one-hybrid experiments showed that OsDREB1D not only can form complexes with proteins that have dehydration response elements/C-repeat motifs, but also can be combined with low-temperature response element (LTRE) sequence. When OsDREB1D was overexpressed in *Arabidopsis thaliana*, the transgenic plants obtained the cold and high-salt tolerance, and were also insensitive to ABA. These results indicated that the functions of OsDREB1D may be similar functions to those of other CBF/DREB1 transcription factors, and that the expression of OsDREB1D in rice may be controlled by a special mechanism for the redundancy of function [52].

Genomic duplications are the essential force driving the origin and evolution of species. However, across evolutionary history, most of these duplications have disappeared or have been silenced, with the remaining few playing a strong role in positive or purifying selection [53]. The substitution rate, known as the Ka/Ks (non-synonymous/synonymous) ratio, is an indicator for estimating selective pressure [54], and is commonly used to analyse the molecular evolutionary rate, and to investigate the evolutionary direction and selective strength of a coding sequence [55]. In this study, nearly every gene pair was subject to purification selection (Ka/Ks < 1), and only one gene pair had a Ka/Ks value greater than 1 in *L. sativa*, experienced positive selection pressure in evolution (Additional file 1b) [53]. The number of CBF/DREB1 proteins and duplication events suggested that the increase in CBF/DREB1 proteins may be due to numerous duplication events in angiosperms. Plants evolve with changes in the environment, and positive selection promotes the exchange of gene functions to survive. Therefore, selective patterns can partially explain the evolutionary patterns of genes [42].

The use of phylogenetic tree is an established method for the examination of the structure and function of a protein family, and the functional relationship can be understood by the phylogeny [56]. We specifically labelled the CBF/DREB1 proteins of potato, tomato, *Arabidopsis* and rice. *Arabidopsis* and rice are common model crop species used in research (Fig. 2). Tomato and potato are related species, and potato is our main research species. The role of a gene in potato can be inferred based on the function of that gene in *A. thaliana*, in tomato, or in rice. For example, overexpression of the AtCBF1 gene in *Arabidopsis* can induce the expression of the COR gene and increase the freezing tolerance of non-acclimated *Arabidopsis* plants [16]. Overexpression of the AtCBF3 gene of *Arabidopsis* in potato can improve the heat resistance of potato by regulating the expression of many genes involved in resistance, increasing the accumulation of soluble substances, improving the photosynthetic performance and antioxidant capacity, and reducing and eliminating excess ROS [57]. In rice, when plants are exposed to low temperatures, the transcripts of OsCBF2, OsCBF8 and OsCBF9 in the roots increase rapidly, and the expression level of OsCBF2 in the leaves increases under salt stress, suggesting that these genes may be involved in the low-temperature response or salt-stress response [50]. As a result, these CBF/DREB1 proteins in potato and tomato might have a similar function or a similar effect on development and responses to various stresses and stimuli.

All BoCBF genes in kale respond to abiotic stresses [49]. In addition, all CBF/DREB1 genes in Chinese cabbage are highly upregulated during cold (4 °C) treatment, and some of them are also responsive to salt (250 mM NaCl), drought (air drying), and ABA (100 mM) treatments [43]. GmDREB1B;1 can activate the expression of numerous soybean-specific stress-responsive genes and enhance ABRE (ABA response element)-mediated gene expression in an ABA-independent manner under a variety of abiotic stress conditions [58]. Therefore, we can also speculate on the function of the corresponding CBF/DREB1 gene in various species based on the existing results, which can provide a clear idea and direction for research.

On the basis of the data obtained from the PGSC database, we measured and analysed the expression levels of the 8 CBF/DREB1 genes at different stages of potato development and under various stresses [59]. The data related to the CBF/DREB1 genes from the PGSC database were analysed in double haploid potato material (DM) or heterozygous diploid material (RH) [45]. The change in the expression levels of the CBF/DREB1 proteins in potato under abiotic stress was different from the results of other studies on CBF/DREB1 genes [43, 49, 50, 57, 58]. The different results may be related to the different species or genotypes uesd. The 10 treatments also did not include cold stress. Therefore, to better understand the response of CBF/DREB1 proteins in potato to temperature stress, we analysed the expression patterns of the eight aforementioned genes of potato under cold (4 °C) and heat (35 °C) stresses using the tetraploid cultivar “Desiree”.

The expression profile analysis showed that under temperature stress, the expression levels of all 8 CBF/DREB1 proteins in potato changed, and the change in transcript levels was also different in different tissues. However, Fig. 6 shows that all genes responded to temperature variations in the leaves, stems, and roots. Interestingly, the expression levels of StCBF3 and StCBF4 significantly increased in the leaves, stems, and roots at 35 °C, which indicated that StCBF3 and StCBF4 were sensitive to high temperature and are worthy of further study.

The DREB subfamily consists of six subgroups [10]. In previous studies, it was found that the StCBF1 gene of the A1 subgroup in potato could improve the cold and freezing tolerance of *Arabidopsis* [60]. The StDREB2 gene of the A2 subgroup was confirmed to be involved in the plant response to several treatments (salt, ABA, etc.) [61]. The StDREB gene of the A4 subgroup plays an important role in the fungal invasion response, and can also improve salt tolerance in potato [62, 63]. Overexpression of the StDREB1 and StDREB2 genes in potato, neither of which is not a member of the A1 subgroup, improves oxidative stress damage by improving plant growth, and proline and antioxidant production, thereby increasing tolerance to cadmium (Cd) [64]. In addition, in other species, the CBF/DREB1 gene plays a very important role in improving plant stress resistance [28, 29, 31, 43, 57, 58]. However, to date, the CBF/DREB1 gene has not been studied in potato. This paper analysed the gene expression patterns of the members of the A1 subgroup of the DREB family in potato, providing an improved theoretical basis and data to further explore the heat tolerance of plants.

## Conclusions

We performed an evolutionary analysis of the CBF/DREB1 protein family in the plant kingdom to reveal the gene structure, phylogenetic relationships, and evolution of the CBF/DREB1 proteins in each group. We identified 292 CBF/DREB1 proteins from 43 species, and CBF/DREB1 proteins were found only in angiosperms. Moreover, the genes of group I and group II were found in only monocots. All the genes of groups II-V were found in eudicots. Therefore, we can speculate that the function of these genes is essential for angiosperms. There were no genes of group I identified in eudicots, therefore, we conclude that group I genes play a crucial role in monocots. We also analysed the gene mapping, motif, gene duplication event, phylogenetic tree, GO annotation and expression data from the PGSC website. These results are important for understanding the properties and functions of CBF/DREB1 proteins, and provide a basis for studying the evolutionary relationships of CBF/DREB1 proteins in plants. Our analysis also showed that all eight CBF/DREB1 proteins in the leaves, stems, and roots of potato are related to high-temperature and low-temperature stresses. In particular, the expression levels of StCBF3 and StCBF4 in the leaves, stems, and roots were upregulated significantly under high-temperature conditions. These two genes may play an important role in the heat tolerance of potato. The above research results increase our understanding of the evolutionary relationships of the CBF/DREB1 family and lay a foundation for further functional identification of the CBF/DREB1 proteins in potato. In subsequent studies, we will try to determine the function of CBF/DREB1 proteins in terms of the biological processes that affect crop resistance and improve the response to various stresses through molecular biological techniques.

## Methods

### Identification of CBF/DREB1 Genes in Various Species

The genes, proteins, and coding sequences of 42 species were downloaded from the Phytozome (https://phytozome.jgi.doe.gov/pz/portal.html), PGSC (https://solanaceae.plantbiology.msu.edu/pgsc_download.shtml), and the NCBI (https://www.ncbi.nlm.nih.gov) database. Six *Arabidopsis* CBF/DREB1 protein sequences that were obtained from TAIR database (https://www.Arabidopsis.org/) were used as queries to perform a protein search against those of 42 species proteins in the database with a strict E value (< 1e^−10^) requirement by using BLAST 2.6.0 [65]. All candidate CBF/DREB1 protein sequences were screened by using the Conserved Domains Database (CDD) (https://www.ncbi.nlm.nih.gov/cdd/) and were aligned using Molecular Evolutionary Genetics Analysis 7 (MEGA 7) software.

### Sequence analysis of CBF/DREB1 genes/proteins

Information on the length of the protein sequences and chromosome location was obtained from the Phytozome, PGSC, NCBI, and other databases (Additional file 1). Multiple Em for Motif Elicitation website (MEME; https://meme-suite.org/tools/meme) was used to calculate the motifs of the protein sequences, with the number of motifs = 20.

### Phylogenetic tree construction and gene duplication analyses

A phylogenetic tree comprising the members of the CBF/DREB1 family from 43 species was constructed using MEGA 7 software. The phylogenetic trees were produced using the neighbour-joining (NJ) method with the parameters of the Jones-Taylor-Thornton (JTT) model and 1000 replicates for bootstrap analysis [66]. The EvolView online tool (https://www.evolgenius.info/ evolview/#login) was used to draw and manage the phylogenetic trees.

Gene tandem duplication events of the CBF/DREB1 genes were analysed following the methods of Gu et al. [67]. The major criterion was that the length of an aligned sequence must cover > 75% of the longer gene, and the similarity of the aligned regions must be > 75%. Two genes were considered a tandem pair if they were located on the same chromosome and were separated by no more than 10 unrelated genes [68]. The segmental duplication pairs were analysed by using the Plant Genome Duplication Database (PGDD; https://chibba.agtec.uga.edu/duplication). Ka and Ks values were calculated by DNAsp software.

### GO annotation and expression pattern analysis

Blast2GO software was used to analyse the Gene Ontology (GO) terms. The full-length amino acid sequences were uploaded into the program, and the NCBI database was chosen as the reference to for analysing the molecular functions, cellular components, and biological processes.

The expression data of the responses of CBF/DREB1 genes to different stresses were obtained from the PGSC database, and the transcript abundance was represented by using fragments per kilobase million (FPKM) values [69].

### Plant materials and growth conditions

The tetraploid potato (*Solanum tuberosum* L.) cultivar “Diseree” was used in this study. Potato plantlets were cultured in MS media supplemented with 10 g/L agar and 20 g/L sucrose (pH 5.8) and were maintained at 25 ± 1 °C under 10,000 lx for 16 h and 20 ± 1 °C under 0 lx for 8 h. After 2 months, we selected seedlings of the same size for experimentation and divided them into three groups. The first group was subjected to a temperature of 35 °C for 24 h, the second group was subjected to a temperature of 4 °C for 24 h, and the third group was maintained under normal conditions as the control. We subsequently sampled the root, stem and leaf tissues (100 mg) from the same parts of the control plants and experimental plants. All of the samples were flash frozen in liquid nitrogen and then stored at − 80 °C prior to utilization.

### RNA extraction and real-time qRT-PCR analysis of CBF/DREB1 gene expression

An RNA simple Total RNA Kit (Code No. RP3301, BioTeke, Beijing, China) was used to extract RNA. The elongation factor 1-a (Ef1a) gene was chosen as the reference gene [70]. Specific primers were designed using Primer Premier 5 software. First-strand cDNAs were synthesized from 1 μg of RNA with a PrimeScript™ RT reagent Kit (Code No. RR047A, TaKaRa, Dalian, China) in conjunction with gDNA Eraser in a 20 μL reaction volume. Real-time PCR was set up on the basis of a 2 × Plus SYBR real-time PCR mixture (Code No. PR7702, BioTeke, Beijing, China) and was performed on a QuantStudio™ 7 Flex Real-time PCR System (Applied Biosystems Inc., America) in a 10 μL reaction volume. The relative expression level of each gene was calculated using the 2^−ΔΔCt^ method [71].

## Supplementary information

**Additional file 1.** Select information about all CBF/DREB1 proteins. a: The protein length, chromosomal location, gene name, and group of each gene. b: Duplication events.

**Additional file 2.** Evolutionary relationships (left), and motif predictions (right) of CBF/DREB1 genes in 43 plant species. The evolutionary history was inferred using the N-J method via MEGA 7. Bootstrap values of 1000 replications were used. The motifs, numbered 1–20, are displayed in different coloured boxes.

**Additional file 3.** Logos of 20 motifs for all CBF/DREB1 proteins.

**Additional file 4.** GO annotation information for each CBF/DREB1 member in potato.

**Additional file 5.** Specific primers used for qRT–PCR analysis.

**Additional file 6.** RNA-seq data of CBF/DREB1 proteins in potato from PGSC database.

## Data Availability

All data generated or analyzed during this study are included in this article.

## Abbreviations

CBF/DREB1: C-repeat binding factor/Dehydration responsive element-binding 1
TFs: Transcription factors
AP2: APETALA2
ERF: Ethylene response factor
RAV: Related to ABI3/VP1
GO: Gene Ontology
BAP: 6-Benzylaminopurine
IAA: Indole-3-acetic acid
ABA: Abscisic acid
GA3: Gibberellic acid
BABA: DL-β-amino-n-butyric acid
BTH: Acibenzolar-S-methyl
